# Supplementary material for: Preconception care uptake and risk factors for adverse pregnancy outcomes among pregnant women in Tigray, northern Ethiopia: A community-based cross-sectional study
Source: PLoS One. 2025 Nov 7;20(11):e0336255. doi: 10.1371/journal.pone.0336255 (PMC12594428; doi:10.1371/journal.pone.0336255)
Supplement: S1 Appendix — (DOCX) [file pone.0336255.s003.docx]

**Uptake of PCC among Pregnant women _tool**

**Annexe-1: consent information sheet and informed consent form a. Participant information sheet**

Hello -------------------------------------------My name is --. I am working temporarily as a data collector with Mekelle University College of Health Sciences, School of Public Health, in a study titled ***"Preconception Care: Closing the Gap in the Continuum of Maternal Health Care in Tigray".*** Duration of the interview: the interview will be conducted in private and taken up to 35 minutes. Purpose: ***The aim of this research is to assess the burden of risk factor for adverse pregnancy outcomes, and determine the uptake of preconception care***. This study will be used as a road map for policymakers and program officers to set priorities, mobilize resources, and develop plans and activities for implementing the new evidence-based preconception care service as a package to improve maternal and perinatal health. Confidentiality: The information collected in this research project will be kept confidential. The questionnaire used to interview you will be kept out of the reach of unauthorized persons, and your name will not be written on it.

The principal investigator will use codes during the data collection period instead of using names. The results of this

study may be used for research publications or presentations at scientific meetings. However, your personal results as an individual will never be discussed with anyone else. Your participation in this research is voluntary. You may choose not to participate, and you may withdraw your consent to participate at any time without losing any of your rights.

Autonomy: Your participation in the study is on a purely voluntary basis. During the interview period, if you find it inconvenient, you have the full right to refuse to take part or interrupt the interview at any time. Your honest and genuine participation in responding to the questions prepared is very important and highly appreciated. Benefits and incentives: There will not be monetary benefits or any special tangible incentives or rewards for your participation in this research project. Risk: The proposed research does not cause any physical harm, social discrimination, psychological trauma, or economic loss. Results Dissemination: The findings of this research will be disseminated to concerned bodies by the researcher to provide feedback to the district health office and the Tigray regional health bureau. Person to Contact: The participant has the right to ask for information that is not clear about the research context and content before or during the research work. You can contact the principal investigator for further information. Principal investigator: Gebremedhin Gebreegziabher Gebretsadik Address: email: [gebremedhingebretsad@gmail.com](mailto:gebremedhingebretsad@gmail.com) Mobile: +251914381043

# General information

Code

location

latitude (x.y °)


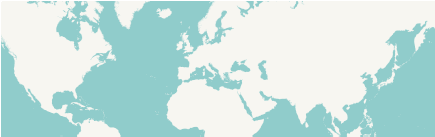

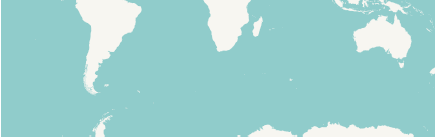


longitude (x.y °)

altitude (m)

accuracy (m)

Name of data collector

phone number of data collector

Zone


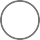

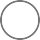
 Central Eastern

Woreda


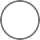
 Adwa district
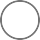
 Ahferom


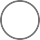
 Adwa town


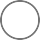

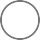
 Tsirae wenberta
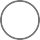
 Klite awlaelo

Wukro town

Date of data collection

yyyy-mm-dd hh:mm

# Section 1: Scio demographic variables

Q101. Current maternal age in completed years

Q102. Place of residence


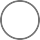
 Rural
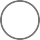
 Urban

Q103. Are you able to read or write simple sentence?


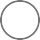
 No
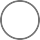
 Yes

Q 104. What is the highest level of schooling you have completed?


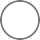
 No education


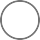
 Primary education(1-8)


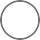
 Secondary and above(9-12)
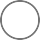
 College and above

Q105. What is your partner/husband education?


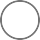
 No education


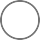
 Primary education(1-8)


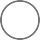
 Secondary and above(9-12)
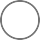
 College and above

Q106.What is your partner's estimated age?

Q107. Length of the current relationship (in years)

Q108. How old were you when you first married?

Q109. What is your total family size in number

Q110. What is your occupation?


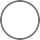
 House wife
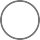
 Self-employed
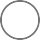
 Merchant


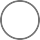
 Governmental employed
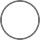
 Farmer


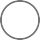
 Student

Q111. What is your partner/husband's occupation?


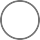
 Farmer


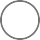
 Self-employed
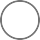
 Merchant


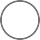
 Governmental employed
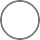
 Daily laborer


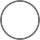
 Student
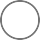
 No work

Q112. What is your religion?


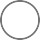
 Orthodox
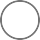
 Muslim
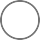
 Catholic
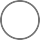
 Protestant

Q113. How long does it take to reach in minutes to the near government health facility on foot?

Q114. Do you have a radio/television in your household?


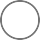

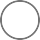
 No Yes

# Section 2: Women's decision making on MCH services

Q201. Who usually makes the decision to obtain health care for you?


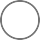
 Myself


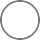
 Women and husband (jointly)
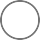
 Women and other person
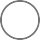
 Husband/partner alone

Q202. Who usually makes the decision for large household purchases


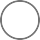
 Myself


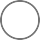
 Women and husband (jointly)
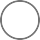
 Women and other person
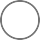
 Husband/partner alone

Q203. Who usually makes the decision during visits to family or relatives?


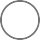
 Myself


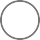

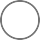
 Women and husband (jointly)
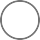
 Women and other person

Husband/partner alone

Section 3: Community health engagement related variables

Q301. Have you ever heard of a model family?


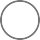
 No
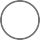
 Yes

Q302. Have you attended pregnant women forum?


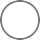
 No
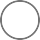
 Yes

Q303. Have you ever discussed about pre pregnancy care in the pregnant women's forum?


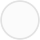
 No
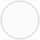
 Yes

Q304. Are you a member of community health insurance


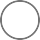
 No
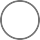
 Yes

Q305. Are you a member of Women Development Group


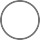
 No
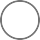
 Yes

Q306. In the past 12 months, have you received any orientation or education from a HEW regarding pre-pregnancy care services?


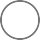
 No
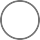
 Yes

# Section 4: MCH services related variables

Q401. How many pregnancies have you had to date?

Q402. How many births have you had to date?

Q403. How many live births have you had up to now?

Q404. How many stillbirths have you had up to now?

Q405. How many neonatal deaths have you had up to now

Q406. How many abortions have you had in your lifetime?

Q407. Have you utilized antenatal care services for the current pregnancy?


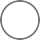
 No
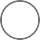
 Yes

Q408. Did you receive an ultrasound (U/S) service for your current pregnancy?


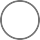
 No
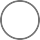
 Yes

Q409. At what gestation age did you receive the ultrasound services?

Q410. What was the indication for ultrasound services?


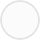
 Routine services
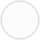
 High risk pregnancy
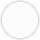
 Emergency

Q411. Where did you receive the ultrasound service?


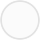
 Health centers
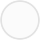
 Hospital


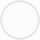
 Private health facilities
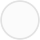
 Health post

Q412. Have you ever used family planning?


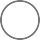
 No
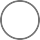
 Yes

Q413. Have you used family planning before current pregnancy?


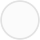
 No
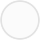
 Yes

Q414. Which types of contraceptives did you used?


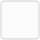
 IUCD


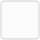
 Injectable
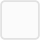
 Implants
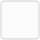
 Pills


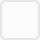
 Male condom


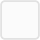
 Emergency contraception
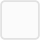
 Breast feeding

Q415. Where did you obtain the current method of contraceptive?


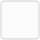
 Health post
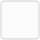
 Health center
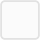
 Hospital


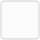

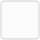
 Private clinic/hospital Private pharmacy

Q416. What was the reason for not used contraceptive?


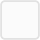
 Side effect


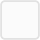
 Lack of availability
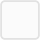
 Lack of information
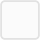
 Need to be pregnant


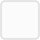
 Opposition from husband
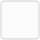
 Opposition from religious


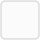
 Opposition from mother-in-law
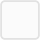
 Shortage of money

# Section 5 : Assessment of Preconception Risk

Q501. Do you have a history of infertility or subfertility within 12 months of attempting pregnancy? (For women under the age of 35)

No Yes

Q502. Do you have a history of infertility or subfertility within 6 months of attempting pregnancy? (For women aged 35 and above

No Yes

Q503. How long is the pregnancy interval between your current and previous pregnancy?

Q504. Do you have a history of any of the following adverse pregnancy outcomes? (Multiple responses are possible)

Pregnancy induced hypertension Ectopic pregnancy

Antepartum hemorrhage Postpartum Depression Obstruct labor Postpartum hemorrhage Rh incompatibility Abortion/miscarriage Congenital anomaly

No Yes

Still birth

Early neonatal death Low birth weight Preterm birth

Late neonatal death Delivery by C-section Oligo-hydramnios

Q505. Do you have a history of any of the following medical problems? (Multiple responses are possible)

Chronic hypertension Diabetes Mellitus

Asthma

Thyroid disorder Epilepsy

Renal disease

Cardiac problem HIV/AIDS

STIs

Hepatitis b virus Cancer

Malaria Anemia

Tuberculosis(TB)

No Yes

Q506. Have you ever smoke any tobacco products, such as cigarettes, cigars or pipes

No Yes

Q507. Do you smoke any tobacco products, such as cigarettes, cigars or pipes currently?

No Yes

Q508. Do you have exposure for at least one hour per day passed in a room, at home, or in a car or in office, with one or more smokers

No Yes

Q509. Have you consumed any type of alcohol, regardless of the amount, during your current pregnancy?

No Yes

Q510. Have you stopped drinking alcohol based on advice from a healthcare provider, such as concerns about negative impacts on your pregnancy outcomes?

No Yes

Q511. Have you ever taken coffee

No Yes

Q512. Do you take coffee currently?

No Yes

Q513. The amount of coffee intake in cup per day

Women dietary diversifications (using 24 hours of food intake)

Q514. 1 Have you taken Grains, white roots and tubers, and plantains

No Yes

Q514. 2 Pulses (beans, peas, and lentils)

No Yes

Q514. 3 Nuts and seeds

No Yes

Q514. 4 Dairy

No Yes

Q514.5 Meat, poultry and fish

No Yes

Q514.6 Eggs

No Yes

Q514.7 Dark green leafy vegetables (Spinach, and salad)

No Yes

Q514.8 Other vitamin A-rich fruits and vegetables (Apple, banana, orange)

No Yes

Q514.9 Other vegetables

No Yes

Q514.10 Other fruits

No Yes

Q515. Do you currently take folic acid supplementation with iron or alone?

No Yes

Q516. 1 Does your work involve a vigorous-intensity activity that causes large increases in breathing or heart rate for at least 10 minutes continuously?

No Yes

Q516.2 In a typical week, on how many days do you do vigorous-intensity activities as part of your work?

Q516.3 How much time in minutes do you spend doing vigorous-intensity activities at work on a typical day?

Q516.4 Does your work involve a moderate-intensity activity that causes small increases in breathing or heart rate for at least 10 minutes continuously?

No Yes

Q516.5 In a typical week, on how many days do you do moderate-intensity activities as part of your work?

Q516.6 How much time do you spend doing moderate-intensity activities at work on a typical day?

Q516.7 Have you walked or use a bicycle (pedal cycle) for at least 10 minutes continuously to get to and from places?

No Yes

Q516.8 In a typical week, on how many days do you walk or bicycle for at least 10 minutes continuously to get to and from places?

Q516.9.How much time in minutes do you spend walking or bicycling for travel on a typical day?

Q516.10 How much time in minutes do you usually spend sitting or reclining on a typical day?

ናይ ህራስ */* ድቃስ */* ግዜ ኣየካትትን

Q517. Have you been exposed to hazardous environmental & household conditions in your day-to-day activities? (Multiple responses are possible)

Do you use an open/traditional type of stoves

Do you use a coal/fuel stove for heating?

Is there any ventilation during heating/cooling?

Do you use a separate cooking kitchen?

Have you undergone X-ray/radiation therapy?

. Have you been exposed to organic solvents (chemicals used in industries or installations like benzene, methanol)

Have you been exposed to pesticides (Insecticides, herbicides and fungicides)

Do you have contact with fertilizer

Q518. During the time of current

pregnancy, how often does your partner? Physically hurt you

Insult or talk down to you Threaten you with harm Scream or curse at you

Q523. What is the updated immunization status? Circle more than one response

Td1

Td2& above

Hepatitis B HPV vaccine Covid -19

No Yes

Never Rarely Often Frequently Always

No Yes

Q519. Do you currently (during

pregnancy) use any of the following medications? (Multiple response is possible)

Taking prescribed medications

**Use self-medication**

Herbal or natural medications

Over the counter medications (aspirin)

Weight- loss medications, and athletic products or supplements

No Yes

Q520. Do the women have anorexia (eating disorder) ?

No Yes

Q521. Do you typically get insufficient sleep, averaging less than 6 hours per night?

No Yes

Q522. Did you know your anemia status during your current pregnancy?

No Yes

Q523. Have you been screened for cervical cancer at least once within the past 5 years?

No Yes

Q524. Did you test for HIV for this pregnancy?

No Yes

Q525. Did you test for hepatitis b during your current pregnancy?

No Yes

Q526. Genetic risks (If yes to any of the queries in the screen)

Do you, your partner, previous children or other relatives have a birth defect,

genetic condition, developmental delay or learning disability?

No Yes

Have you had two or more miscarriages history?

Have you or your partner had a previous pregnancy end because of a birth defect, genetic disease, or death before or after birth?

Will you be 35 years old or older when you plan to give birth?

Q527. Do you currently have any of the following signs of periodontal disease? (Multiple response is possible)

Red or swollen gums Tender or bleeding gums Painful chewing

Loose teeth (tooth decay) Dental or gum pain

No Yes

Q528. The questions to assess psychological distress using (K10) About your feelings and thoughts about you over the past four weeks and the response options are: 1 = none of the time, 2 = a little of the time, 3 = some of the time, 4 = most of the time, and 5 All of the time.

About how often did you feel tired out for no good reason?

About how often did you feel nervous?

About how often did you feel so nervous that nothing could calm you down?

About how often did you feel hopeless?

About how often did you feel restless or fidgety?

About how often did you feel so restless you could not sit still?

About how often did you feel depressed?

About how often did you feel that everything was an effort?

About how often did you feel so sad that nothing could cheer you up?

About how often did you feel worthless?

none of the time

a little of the time

some of the time

most of the time

All of the time

Q529. Are you abstaining from animal-based foods, such as meat, dairy products, and eggs, for religious reasons every Wednesday and Friday throughout the year, including during longer fasting periods?

No Yes

# Section 6: Planning of pregnancy related factors

Q601. Did you used contraceptive at the time of conception (contraception)

Always used contraception Inconsistent use

Not using contraception

Q602.What was your personal circumstances in terms of time becoming a mother(timing)

Wrong time

OK but not quite right Right time

Q603. Did you have an intention of pregnancy before conception(intention)

Did not intend to become pregnant Changed intentions

Intended to get pregnant

Q604. Do women have a desire for pregnancy just before conception (desire

Did not want a baby

Mixed feelings about having a baby Wanted a baby

Q605. Did you have partner influences before conception (partner)

Had never discussed children Discussed but no firm agreement Agreed pregnancy with a partner

Q606. Did you have pre-conceptual preparation (FA, seeking health advice) before conception(preparation)

Did no preparatory life style change Did 1 preparatory life style change

Did 2 or more preparatory life style change

# Section 7: Attitude of women on preconception care

Q700. Respond the attitude questions towards PCC, and the response options

are: 1= strongly disagree, 2 = disagree, 3 = neutral, 4= agree, and 5 = strongly agree

1. PCC is a high health care priority for all women/couples planning pregnancy
2. Women with medically confirmed diseases should only receive PCC services

strongly disagree

disagree neutral agree strongly

agree

1. Women who had pregnancy complications or adverse birth outcomes previously should only receive PCC services
2. PCC services should be provided for reproductive age women
3. PCC services should be provided for married women
4. PCC services should be provided for unmarried women
5. PCC services are not important for adolescent girls
6. Husbands should accompany their wives while seeking any PCC services
7. PCC services should be provided by male health professionals
8. PCC services should be provided by female health professionals.
9. PCC services should be provided by traditional birth attendants
10. PCC does not have any effect on birth outcomes

# Section 8: Uptake of preconception care services

Q801. Did you make any preparation for the current pregnancy before conception?

Nothing do

prepared for myself at home Consult healthcare provider Visit to traditional healers

**Q802. Have you ever heard about pre-pregnancy care?**

No Yes

**Q803. If your response for Q 802 is** where **did acquire the information you**

**hear?**

Health professionals

Mass media

Family or friends

Leaflets or broachers

School/college

804. Have you received preconception care service (like screening or counseling or treatment from healthcare provider) in the current pregnancy before conception?

No Yes

Q 805. As part of preconception care, did you receive any of the care (screening, or counseling or treatment) listed below from healthcare providers?

Iron-folic acid supplementation

Psychosocial support (depression anxiety and stress)

No Yes

prevention of substance use(alcohol, smoking, intake of coffee above optimal, chat)

Family planning and contraception

Nutritional counseling(weight measurement, diet practice, and anemia)

Infectious disease(HIV, STI, Hepatitis, malaria and TB)

Chronic medical disease(diabetic mellitus, hypertension, kidney problem, Thyroid disorder, asthma,

epilepsy and Cardiovascular disease ) Physical activities

Vaccine preventable disease(vaccinate the recommended vaccine, Td )

Genetic risks

Medicine with adverse outcomes (effect of medication on pregnancy)

Gender biased violence Dental health

Exposure to Environmental or household risks

Reproductive organ anomalies and cervical cancer

Female genital mutilation

Q806.What barriers prevented you from seeking PCC services? (Multiple responses are possible)

Respondent didn't think necessary Unavailability of the services

Did not know where to go Unplanned pregnancy

Lack of awareness in providing PCC in health facilities Long distance to PCC health facilities

lack of money

# Section 9: Risk perception questions for preconception risks

Q901. Answer the following questions about the perception of preconception risks by selecting the appropriate scores: 1 = Definitely not, 2 = Probably not, 3 = I am not sure, 4 = Probably yes, 5 =

Definitely yes

*Perceived susceptibility to preconception risks*

Do you believe that you are at a risk for preconception risk factors for APOs

Do you believe that preconception risk is a significant concern for women in your community?

Are you currently worried about the problem of preconception risks?

Do you consider yourself that it is less likely to be a risky for preconception risks in the future

Definitely not

Probably not

I'm not sure Probably yes Definitely yes

Q902.Answer the following questions about the perception of preconception risks by selecting the appropriate scores: 1 = Definitely not, 2 = Probably not, 3 = I am not sure, 4 = Probably yes, 5 =

Definitely yes.

*Perceived se verity to preconception risks*

Do you believe that preconception risks are a potentially fatal?

Definitely not

Probably not I am not sure Probably yes Definitely yes

Do you believe that preconception risks will result in long-term health consequences for both women and newborn baby?

Do you believe that a preconception risk extremely poses a significant threat to health women and newborn baby?

Are you concerned about the potential effect associated with preconception risks if they are not identified early?

Are you much concerned about the potential adverse pregnancy outcomes of preconception risks?

# Section 100: MUAC measurement

Q101. What is the value of pregnant women’s MUAC?
