# Supplementary material for: Preconception care uptake and risk factors for adverse pregnancy outcomes among pregnant women in Tigray, northern Ethiopia: A community-based cross-sectional study
Source: PLoS One. 2025 Nov 7;20(11):e0336255. doi: 10.1371/journal.pone.0336255 (PMC12594428; doi:10.1371/journal.pone.0336255)
Supplement: Attachment — Submitted filename: Response to Reviewers.docx [file pone.0336255.s005.docx]

**Date: September 15 2025**

**Manuscript Number:** PONE-D-25-35031

**Title:** **Preconception Care Uptake and Risk Factors for Adverse Pregnancy Outcomes among pregnant women in Tigray, northern Ethiopia: A Community-Based Cross-Sectional Study**

By: Gebremedhin Gebreegziabher Gebretsadik^1, 2*^, Andargachew Kassa  Biratu^4^,Alemayehu Bayray Kahsay ^2^, Amanuel Gessessew ^3^, Zohra S. Lassi, ^5, 6^_,_ Hailemariam Segni^7^ , Afework Mulugeta ^2^

To: PLOS one

Dear Editor,

We, the authors of this manuscript, express our gratitude to the journal editors and reviewers for their thorough review, insightful comments, and valuable suggestions that significantly enhanced the quality of our work. We have rigorously revised the manuscript as per your questions and comments. We have included the point-by-point response in the table below, framed as **Editor(s)’and Reviewers' comments** and authors' responses. The detailed revisions and changes we made in the main document are prepared with track changes attached separately. We expect our revision will enable the manuscript to fit the journal better. Should you have any further inquiries, please feel free to contact us at your convenience.

**Point-by-point Responses to Editor’s and Reviewers Comments**

| **Editors and Reviewers' comments** | Authors’ Response |
| --- | --- |
| **Editor comments** |  |
| 1. Please ensure that your manuscript meets PLOS ONE's style requirements, including those for file naming. | Thank you very much, we did it accordingly. |
| 2. Please amend either the abstract on the online submission form (via Edit Submission) or the abstract in the manuscript so that they are identical. | Thank you for your valuable suggestion. We have accepted your comment and made the correction. |
| 3.  We note that Figure 1 in your submission contain [map/satellite] images which may be copyrighted. All PLOS content is published under the Creative Commons Attribution License (CC BY 4.0), which means that the manuscript, images, and Supporting Information files will be freely available online, and any third party is permitted to access, download, copy, distribute, and use these materials in any way, even commercially, with proper attribution. For these reasons, we cannot publish previously copyrighted maps or satellite images created using proprietary data, such as Google software (Google Maps, Street View, and Earth). For more information, see our copyright guidelines: <http://journals.plos.org/plosone/s/licenses-and-copyright>.  We require you to either (1) present written permission from the copyright holder to publish these figures specifically under the CC BY 4.0 license, or (2) remove the figures from your submission:  1. You may seek permission from the original copyright holder of Figure 1 to publish the content specifically under the CC BY 4.0 license.  We recommend that you contact the original copyright holder with the Content Permission Form (http://journals.plos.org/plosone/s/file?id=7c09/content-permission-form.pdf) and the following text:  “I request permission for the open-access journal PLOS ONE to publish XXX under the Creative Commons Attribution License (CCAL) CC BY 4.0 (http://creativecommons.org/licenses/by/4.0/). Please be aware that this license allows unrestricted use and distribution, even commercially, by third parties. Please reply and provide explicit written permission to publish XXX under a CC BY license and complete the attached form.”  Please upload the completed Content Permission Form or other proof of granted permissions as an ""Other"" file with your submission.  In the figure caption of the copyrighted figure, please include the following text: “Reprinted from [ref] under a CC BY license, with permission from [name of publisher], original copyright [original copyright year].”  2. If you are unable to obtain permission from the original copyright holder to publish these figures under the CC BY 4.0 license or if the copyright holder’s requirements are incompatible with the CC BY 4.0 license, please either i) remove the figure or ii) supply a replacement figure that complies with the CC BY 4.0 license. Please check copyright information on all replacement figures and update the figure caption with source information. If applicable, please specify in the figure caption text when a figure is similar but not identical to the original image and is therefore for illustrative purposes only.  The following resources for replacing copyrighted map figures may be helpful:  USGS National Map Viewer (public domain): http://viewer.nationalmap.gov/viewer/  The Gateway to Astronaut Photography of Earth (public domain): http://eol.jsc.nasa.gov/sseop/clickmap/  Maps at the CIA (public domain): https://www.cia.gov/library/publications/the-world-factbook/index.html and https://www.cia.gov/library/publications/cia-maps-publications/index.html  NASA Earth Observatory (public domain): http://earthobservatory.nasa.gov/  Landsat: http://landsat.visibleearth.nasa.gov/  USGS EROS (Earth Resources Observatory and Science (EROS) Center) (public domain): http://eros.usgs.gov/#  Natural Earth (public domain): http://www.naturalearthdata.com/ | Thank you for your comment. The study area map was created by our team using administrative boundary shapefiles for Ethiopia (regions, zones, and woredas) downloaded from DIVA-GIS ([www.diva-gis.org/data](https://www.diva-gis.org/data)). This data was processed in GIS software to produce the original map. The resolution was then adjusted to 300 DPI to meet PLOS ONE's publication standards. We confirm the map is an original creation and was not sourced from Google Maps, Earth, or Street View. |
| 4. Please include captions for your Supporting Information files at the end of your manuscript, and update any in-text citations to match accordingly. Please see our Supporting Information guidelines for moreinformation:<http://journals.plos.org/plosone/s/supporting-information>. | Thank you. We duly accept your comment and have considered it. We included file data set and the questioner at the end of a manuscript as supporting information (page 31, lines 568 to 571) |
| 5.  We are unable to open your Supporting Information file [PCC data_3.sav].  Please kindly revise as necessary and re-upload. | Thank you for your suggestion. The SPSS dataset is functioning correctly in my system even after being downloaded from the file sent to the journal. However, we have reuploaded the updated dataset in both Excel/CSV and SPSS formats as part of the supporting information. |
| 6. In the ethics statement in the Methods, you have specified that verbal consent was obtained. Please provide additional details regarding how this consent was documented and witnessed, and state whether this was approved by the IRB. | Thank you for your valuable comment. We have incorporated this feedback into the document. As requested, we have stated: "After fully explaining the study's objectives, risks, and benefits, verbal consent was obtained from all participants. This verbal of consent was approved by the Institutional Review Board of Mekelle University, College of Health Sciences." For better detail, this statement has been included in the Ethical Considerations section of the manuscript (page 15, lines 320 to 323). |
| 7. If the reviewer comments include a recommendation to cite specific previously published works, please review and evaluate these publications to determine whether they are relevant and should be cited. There is no requirement to cite these works unless the editor has indicated otherwise. | Thank you for your kind suggestion. |
| **Reviewer #1:** |  |
| **Abstract:** |  |
| 1. The method section needs more details such as how participants selected using interviews? And authors did not write that they conducted the study among pregnant or non-pregnant women. The instruments and outcomes are so important, and authors should mention these two factors. | Thank you for your valuable perspective. Your suggestion has been duly accepted, and we have incorporated the modifications into the methods section of the abstract (page 2, liness 28–33). |
| 2. The participants were in which gestational age, and were they primipara or multipara? | Thank you again for your comment. The study included pregnant women in their first or second trimester to enhance recall accuracy and minimize potential recall bias. Participants were enrolled regardless of gravida status, including both primiparous and multiparous women. |
| 3. What authors mean about PCC service uptake was 7.2%? Is that mean that only 7.2% of women used PCC? | Thank you for your request for clarity. The overall uptake of PCC services among the 764 pregnant women was low, at only 7.2%; this corresponds to 55 women who received these services. |
| 4. Please write the conclusion according to your results. In this study you did not evaluate the knowledge. Only focus on your objectives. | We assessed awareness of preconception care (PCC) services and found it to be very low, with only 12.2% of pregnant women being informed about these services. Our analysis also demonstrated that awareness had a statistically significant relationship with improved PCC uptake. Additionally, study participants identified lack of awareness (31.9%) as a primary barrier to service utilization. For these reasons, we have included awareness improvement as issues that needed to be addressed in order to enhance PCC uptake. Over all we modified the conclusion of the abstract and included in the manuscript (page2to 3, lines 41 to 44) |
| **Introduction** |  |
| 1. What is HSTP II? Please write each word in full before using its abbreviation. | Thank you very for comment and we duly accept and made a correction in the introduction part of the manuscript which HSTP II means Health Sector Transformation Plan II (Page 4, line 84) |
| 2. Please write about the current situation of PCC in Ethiopia? Is PCC mandatory for women of reproductive age? and if so, who is responsible to do this care in public health centers? | Thank you again for the valuable and meaningful insight.  Preconception care (PCC) is globally recognized as an essential component of maternal healthcare, endorsed by leading health authorities like the World Health Organization (WHO) and the Centers for Disease Control and Prevention (CDC) (1). This preventive approach is designed to optimize women's health before pregnancy to reduce adverse outcomes.  While some nations, such as China, have successfully integrated mandatory PCC programs, Ethiopia continues to face significant challenges in maternal and child health. Despite decades of improvement, the country's progress remains insufficient to meet its Sustainable Development Goal targets.  The effectiveness of preconception care is well-documented (2). Despite these proven benefits, uptake remains low across Sub-Saharan Africa; a trend consistent with the situation in Ethiopia. Evidence demonstrates that PCC can significantly reduce adverse pregnancy outcomes (3). including lowering the rate of birth defects from 5.6% to 2.5% (4).  Despite these proven benefits, uptake remains low across Sub-Saharan Africa (5), a trend consistent with the situation in Ethiopia (6).  Recognizing this gap, the Ethiopian government has made notable efforts to institutionalize PCC. The Ministry of Health adopted a policy on PCC in 2020 and subsequently developed national guidelines to implement the program across all tiers of the health system, down to the community level, via trained healthcare providers (7). PCC is a key component of the national five-year Reproductive Health Strategy (2020–2024/25), which aims to reach 25% of pregnant women by 2025. The policy mandates that all women of reproductive age and couples with pregnancy intention should access PCC services (8).  Additionally, the recent pooled prevalence of a good level of preconception care (PCC) practice among Ethiopian healthcare providers was 53.54%(9), indicating promising progress in the country's health facilities.  However, based the report of this study in Tigray showed that only 7.2% of pregnant women were received the PCC and only 12.2% of them had information about the services suggests that implementation on the ground is low.  In conclusion, while Ethiopia has demonstrated strong policy commitment to preconception care as a flagship program to reduce the high burden of adverse pregnancy outcomes, a significant disparity exists between policy intent and practical implementation. As PCC services are included in the maternal continuum of care to tackle the high rates of maternal and newborn mortality and morbidity, a concerted effort is needed to bridge this gap.  We included a general overview of preconception care (PCC) as a national program in Ethiopia in the introduction section of the manuscript (page 5, lines 88 to 90). |
| 3. How many visits are considered as optimal for PCC? | Thank you for this valuable insight. It is recommended that women with the potential to become pregnant receive at least one PCC visit, (10) with additional visits as needed based on individual risk factors. Included in the method section of a manuscript(page 9, lines 191 to 192) |
| 4. Is PCC active in your region? For instance, if the woman does not come to the health center, a health provider, will call them? | According to the adopted national policy of Ethiopia, all women of reproductive age who intend to become pregnant within the next three to six months should receive preconception care (PCC) services (8), (7). To support this mandate, responsible health providers have been trained to deliver PCC in accordance with the National PCC guideline.  However, the implementation of this program faces significant challenges, particularly in the Tigray region. As a relatively new initiative, PCC has been further compromised by a health system weakened by recent conflict(11). This has shifted immediate health priorities, making it difficult to fully mobilize and promote PCC services at the community level with the same effectiveness as established programs like antenatal care (ANC).  Despite these systemic obstacles, there has been some progress. Certain Health Extension Workers (HEWs) are actively working to mobilize and refer eligible women for PCC. Furthermore, some healthcare providers, particularly midwives and obstetrics/gynecology professionals, are making commendable efforts to practice PCC more consistently than other providers. For example, the recent pooled prevalence of a good level of preconception care (PCC) practice among Ethiopian healthcare providers was 53.54%(9), indicating promising progress in the country's health facilities.  Therefore, while a strong policy and clinical guideline framework exists for PCC, its practical application in Tigray remains inconsistent due to systemic constraints and competing health priorities. The nascent progress, driven by dedicated frontline workers and specialists, demonstrates potential. However, achieving widespread uptake requires targeted support to strengthen the health system, refocus community health priorities, and expand training to all healthcare providers to ensure PCC becomes an integral part of the maternal health continuum.  The overall concept was included in the introduction section of the manuscript (page 5, lines 88 to 90). |
| **Methods** |  |
| 1. What is MUAC? | Thank you for the comment. Mid- Mid-upper arm circumference (MUAC) is a quick and reliable tool for assessing nutritional status and shows a strong correlation with BMI(12). It is already used in adult care in resource-limited settings and for monitoring maternal undernutrition. Evidence from a cohort study in Brazil further supports MUAC as a practical surrogate for BMI in rapidly screening the nutritional status of pregnant women (13). |
| 2. What is the justification for 778 of sample size? | Thank you once again for your comment. The sample size for this interventional study was calculated using Epi-Info software, employing a two-sided Z-test for proportions with 80% power and a 5% significance level(14). Based on a baseline preconception care (PCC) uptake of 16.27% (6) and an anticipated 10% increase post-intervention, the calculation incorporated a design effect of 2.5 to account for cluster sampling and a 20% adjustment for potential non-response. This yielded a required sample size of 778 participants per group. Finally, we included 764 pregnant women with a 98.2% response rate for analysis. These details are provided in the manuscript methods section (page 7 to 8 lines 142 to 151). |
| 3. Measurements of PCC service uptake: please clarify for readers, if women received PCC in few fragments, for instance, in one session they received only screening or counselling, or if they receive PCC in a single package. | Thank you once again for your comment.  The uptake of PCC was measured by determining whether women had consulted a healthcare provider for PCC and had received at least one service from the WHO-recommended package and national PCC guideline before current pregnancy (1)**(15)** (7). Further, uptake was categorized as follows: (1) **no uptake** (no components received); (2) **partial uptake** (at least one component received); and (3) **optimal uptake** (folic acid supplementation plus at least one other component). We included in the method section of a manuscript (page 9, lines 185 to 192). |
| 4. How authors evaluated the validity and reliability of instrument that they used for assessing attitude? | Thank you for your comment. The questionnaire was developed based on a literature review and professional experience, then adapted to the local context. It was pre-tested with 39 pregnant women, and subsequent refinements were made before being deployed on the KoBo Toolbox for data collection. Attitudes toward preconception care were specifically measured using a validated 12-item Likert scale, with responses ranging from "strongly agree" to "strongly disagree" (16). (Page 10, line 198 to 203) |
| 5. Line 190: While authors intended to assess the PCC, why they asked about ultrasound service within the first 24 weeks of gestation? | Thank you for your valuable comment. Ultrasound scanning within the first 24 weeks of gestation is a newly introduced service for all pregnant women in the Ethiopian healthcare system. We assessed its uptake as an independent variable to explore its potential relationship with the uptake of preconception care (PCC) services. This will help guide future interventions. |
| 6. Line 208: What do you mean about street medications? Do you mean home-remedies? | Thank you very much; we greatly appreciate your insight. In this study, 'street medication' refers to the practice of self-medication during pregnancy, where medications are used without health care provider guidance to treat symptoms. We have corrected and included in the methods section of the manuscript (page 11, lines 229 to 230). |
| 7. Line 214: Did you evaluate alcohol drinking only during pregnancy or ever consumption? | Thank you again for your comments. We assessed and reported alcohol consumption only during the current pregnancy. |
| 8. Line 230: Please provide reference for the mid-upper arm circumference of > 23 indicated well nutritional status. | Thank you again for your suggestions. We have accepted your comment and have incorporated the provided reference into the Methods section. The added text is: "Pregnant women with a mid-upper arm circumference (MUAC) of ≥23 cm were classified as well-nourished, and those with a MUAC <23 cm were classified as undernourished (17),(18)“This has been included in method section of a manuscript(page 12, line 252 ) |
| 9. In my opinion, for assessing dietary diversity, authors should have used a better questionnaire, not just asking participants about what they consumed during the past 24 hours. | We sincerely thank the reviewer for their insightful comment regarding dietary assessment methodology. We fully agree on the importance of selecting a valid and reliable instrument.  Our use of the 24-hour recall was a deliberate decision aligned with our objective of assessing population-level dietary diversity. This method is not an oversimplification but rather the internationally recommended tool (per FAO guidelines) for calculating the Minimum Dietary Diversity for Women (MDD-W) score. It is specifically designed as a rapid, cost-effective screening tool to identify micronutrient deficiency risk in populations.  We acknowledge this method's limitation in capturing individual habitual intake due to day-to-day variation and reliance on memory. In an ideal scenario, the interactive 24-hour record (the gold standard for accuracy) would be preferable. However, this method is prohibitively resource-intensive for large-scale studies like ours.  Therefore, we made a deliberate trade-off between precision and feasibility, selecting the most appropriate tool for group-level comparisons. Our instrument was adapted from established protocols (FAO, FANTA, and DHS) to ensure methodological robustness and international comparability. We mitigated recall bias through trained enumerators using structured interview techniques.  We revised the methods section to more explicitly cite these foundational references and included the limitation in the discussion section of the manuscript (page 12, lines 254 to 259 ). |
| 10. What were the inclusion/exclusion criteria? | Thank you again for your suggestions. We have duly accepted your comment and have incorporated the inclusion/exclusion criteria into the Methods section of the manuscript (page 6, lines 123 to 126) |
| 11. How did you classify the attitude to poor and good? | The study measured women's attitudes toward preconception care using a 12-item Likert scale questionnaire. To ensure accuracy, the instrument included both positively and negatively worded statements. The scoring protocol was standardized so that a higher score always reflected a more positive attitude; this was achieved by reverse-coding the responses to negative statements (e.g., 'Strongly Agree' = 5 for positive statements, but = 1 for negative statements). An individual's total attitude score was calculated by summing their responses across all 12 questions, yielding a possible range of 12 to 60 points. This total was then divided by 12 to compute a mean score for each respondent. Finally, for analytical purposes, respondents were classified into two groups: those with scores equal to or above the mean were categorized as having a "positive attitude", while those scoring below the mean were categorized as having a "negative attitude". We included in the method section of a manuscript (page 10, lines 198 to 203) |
| ***Results*** |  |
| 1. Table 1: In my opinion, because many people nowadays follow health instruction via their smart phone, they do not need to have TV or radio. So, it was better that authors asked about smart phone. | We thank the reviewer for their valuable comment regarding the importance of assessing smartphone use for health information access. We agree that smartphones represent an increasingly relevant channel for health communication.  While our study did not explicitly measure smartphone-based health information seeking a limitation we will acknowledge this was in part due to contextual factors. According to the 2019 Ethiopia Demographic and Health Survey, only 18% of women aged 15–49 used the internet, with a pronounced urban-rural disparity. Given that smartphones are the primary means of internet access in Ethiopia, these figures suggest that smartphone availability for health information remained limited and inequitable at the time of data collection, particularly in rural areas.  Furthermore, our data were collected in the Tigray region, where access to all media including smartphones, radio, and television has been severely affected by recent conflict. In such settings, radio has historically offered broader and more equitable reach, though all communication channels remain compromised.  We agree that future studies should include smartphone use where feasible. Thank you again for this insightful suggestion. |
| 2. Line 334: What do you mean about “women reported fasting during their pregnancy”? Is it due to the religious rules or just for diet? | Thank you once again for your valuable comments. Fasting status was measured by asking if participants abstained from animal-based foods (meat, dairy, eggs) for religious reasons on Wednesdays, Fridays, and during extended fasting periods (yes/no) (19)., and incorporated in the methods of a manuscript (page 11, lines 215–217). |
| 3. Table 5; Please explain about physical activity and how did you divide participants into active or inactive? | Thank you very much for sharing your valuable perspective.  Physical activity was assessed using a tool adapted from the International Physical Activity Questionnaire (IPAQ), which captured recreational, occupational, and transport-related activity via a 7-day recall. Participants were categorized into three activity levels—low, moderate, or high—based on established IPAQ guidelines. These categories were derived by calculating weekly MET-minutes, where MET values (walking = 3.3, moderate = 4, vigorous = 8) were multiplied by the duration and frequency of each activity. For example, walking 30 minutes for 5 days yields 495 MET-minutes. Classification was then determined as follows: high activity (≥1500 MET-minutes of vigorous activity on ≥3 days, or ≥3000 MET-minutes from any combination), moderate activity (≥600 MET-minutes, or meeting specific frequency and duration thresholds), and low activity (not meeting moderate or high criteria) (20), Finally, we reported participants as 'active' by merging the moderate and high activity groups, and recoding the low activity group as 'inactive. Fore detailed included in the methods part of a manuscript. we modified and included in the method section of a manuscript (page 13, lines 261 to 271) |
| **Reviewer #2:** |  |
| **Abstract** |  |
| 1. The title should clearly reflect the study’s focus on preconception care (PCC) uptake and pregnant women's experiences in Tigray, Ethiopia. | Thank you for this constructive suggestion. We agree that the title should modify. Accordingly, we have revised the title to: *“****Preconception Care Uptake and Risk Factors for Adverse Pregnancy Outcomes among pregnant women in Tigray, northern Ethiopia: A Community-Based Cross-Sectional Study*** |
| 2. Consider including key elements such as:  * The study design (e. community-based cross-sectional study”) * Study population (pregnant women) * Location and time frame (Tigray, 2024) | Thank again we considered you comment |
| 3.A more informative title could be:  Preconception Care Uptake and Associated Factors Among Pregnant Women in Tigray, Ethiopia: A Community-Based Cross-Sectional Study | Thank you again for your constrictive comment.  Accordingly, we have revised the title to: *“****Preconception Care Uptake and Risk Factors for Adverse Pregnancy Outcomes among pregnant women in Tigray, northern Ethiopia: A Community-Based Cross-Sectional Study*** |
| Methodology |  |
| 1. Consider updating the antenatal care statistics with more recent data if available, as the current reference is from the 2007 census | Thank you for your suggestion. We have updated the total population of the Tigray region in the study setting section (page.5, lines 104–108).  However, the study citing the proportion of antenatal care services was conducted in 2024 and did not use the 2007 population data. The 2007 figure was originally included only to provide a simple demographic context for the region. We have now corrected this by using the updated population data. Regarding population proportion allocation for data collection, we determined the number of pregnant women in each cluster based on the current population estimates for each *woreda* and *kebele* (village) at the time of data collection. |
| 2.Good justification is given for trimester selection, but clarify whether third-trimester women were excluded and why? | Thank you for this helpful comment.  Following the conflict in selected Tigray districts, food aid was provided to pregnant and lactating mothers with a MUAC <23 cm, supported by partners. Although confirming pregnancy status does not ensure ANC access, most women suspecting pregnancy verified their status earlier at health facilities, receiving a confirmation slip for HEWs. Nearly all women knew their pregnancy status before the survey. Additionally, the Tigray Regional Health Bureau and WHO conducted a SMART survey before data collection, involving HEWs to profile pregnant and lactating mothers for targeted interventions. Though have no significant effect level of trimester of the Gestational age of the pregnancy, for the sake of sufficient follow-up time for assessing service utilization and accessible of sufficient number pregnant women who know their pregnancy status relatively early because of the above reason the to support food aid and to minimize recall bias we included first and second trimester to this study. |
| 3.The reliance on HEWs and WDGs may introduce selection bias; briefly acknowledge or address this potential limitation. | Health Extension Workers (HEWs) and Women’s Development Army (WDA) leaders play complementary and critical roles in extending maternal healthcare services to the community level in Ethiopia. HEWs, as trained government health workers, are responsible for providing basic antenatal care including health education, iron-folic acid supplementation, immunizations, and danger sign screening as well as facilitating referrals for emergencies and higher-level care, and maintaining maternal health records. They are supported by unsalaried WDA leaders, who serve as community volunteers and act as a "first alert" system: they identify pregnant women through daily interactions, encourage timely antenatal care registration, provide social support, and promptly report emergencies to HEWs for action. This tiered, collaborative model effectively bridges the formal health system and households, ensuring broader reach and earlier detection of pregnancy-related needs and complications(21).  Of the routine responsibilities identify and register all pregnant women in their catchment to ensure they receive proper prenatal and postnatal care.  However, following the conflict in Tigray, a new process was established for monthly food aid distribution. To receive aid, pregnant women were required to obtain a confirmation slip from HEWs to verify their pregnancy status. Furthermore, a prior SMART survey conducted by the Tigray Regional Health Bureau and WHO involved HEWs in profiling pregnant and lactating mothers for aid targeting.  These two initiatives provided HEWs and WDGs with a unique opportunity to easily identify and register the entire pregnant population. For the study itself, pregnant women were then selected via simple random sampling from these newly compiled lists in each tabia/kebele. |
| 4. Report how the final sample size (764) was calculated or adjusted from the initial 778, and whether any exclusions occurred during data collection. | Thank you once again for your comment. The sample size for this interventional study was calculated using Epi-Info software, employing a two-sided Z-test for proportions with 80% power and a 5% significance level(14). Based on a baseline preconception care (PCC) uptake of 16.27% (6) and an anticipated 10% increase post-intervention, the calculation incorporated a design effect of 2.5 to account for cluster sampling and a 20% adjustment for potential non-response. This yielded a required sample size of 778 participants per group. Finally, we included 764 pregnant women with a 98.2% response rate for analysis. The reason for non-response were time constraints from childcare and chores, and pregnancy-related fatigue or discomfort. Included in the result section a manuscript (page 7 to 8, lines 142 to 151). |
| 5. Mention any pilot testing or pretesting of the questionnaire (even if briefly) to validate tools for local context. | Thank you for this important comment. The questionnaire was pretested in a different zone of the region to ensure its clarity, relevance, and cultural appropriateness. Following modifications based on the pretest feedback, the final version was uploaded to the Kobo Smart Survey platform. This information is included in the data quality assurance part of Methods section of a manuscript (page 14, lines 295 to 302). |
| 6.In Access to Health Services: Clarify if any adjustments were made for terrain, transportation availability, or security issues, especially in post-conflict settings. | We sincerely thank you for raising this important point. In response to concerns about terrain, transportation, and security within northern Ethiopia’s post-conflict setting, we implemented several methodological adaptations during data collection including hiring local guides, and deploying data collectors in teams to minimize bias and enhance access to remote communities. Although no direct statistical adjustments were made for these contextual factors, the self-reported reasons participants provided for not seeking preconception care (PCC) such as the perception that care was unnecessary, unavailability of services, lack of awareness, uncertainty about where to go, and financial constraints, including lack of money for transport are presented in the results. We included this in to the s**tudy design and population section of methods in the manuscript (page 6, lines 119 t0 122)** |
| 7. The PCC components list is comprehensive, but you might consider summarizing or using a table for readability. | We appreciate the reviewer's valuable suggestion. In response, we would like to note that the results section is primarily composed of tables, with two figures. To maintain this structure and clarity, we have chosen to keep the presentation of PCC components within the existing figure. |

Thanks

Reference

1.	Organization WH. Meeting to develop a global consensus on preconception care to reduce maternal and childhood mortality and morbidity: World Health Organization Headquarters, Geneva, 6–7 February 2012: meeting report. 2013.

2.	Boerma T, Requejo J, Victora CG, Amouzou A, George A, Agyepong I, et al. Countdown to 2030: tracking progress towards universal coverage for reproductive, maternal, newborn, and child health. The Lancet. 2018;391(10129):1538-48.

3.	Hood JR, Parker C, Atrash HK. Recommendations to improve preconception health and health care: strategies for implementation. Journal of Women's Health. 2007;16(4):454-7.

4.	Xu J, Li X, Zhou Q. Nationwide-free preconception care strategy: Experience from China. Frontiers in public health. 2022;10.

5.	Woldeyohannes D, Tekalegn Y, Sahiledengle B, Hailemariam Z, Erkalo D, Zegeye A, et al. Preconception care in sub-Saharan Africa: A systematic review and meta-analysis on the prevalence and its correlation with knowledge level among women in the reproductive age group. SAGE Open Medicine. 2023;11:20503121231153511.

6.	Ayele AD, Belay HG, Kassa BG, Worke MD. Knowledge and utilisation of preconception care and associated factors among women in Ethiopia: systematic review and meta-analysis. Reproductive health. 2021;18(1):1-15.

7.	Mo. hE. Ensuring a risk free conception experience! National Preconception care Guidelines. 2024.

8.	Health EMo. Health Sector Transformation Plan II (HSTP II): 2020/21-2024/25 (2013 EFY-2017 EFY). MOH; 2021.

9.	Zeleke AM, Tassew WC, Assefa GW, Ferede YA. Healthcare providers' preconception care practice and associated factors in Ethiopia: a systematic review and meta-analysis. Frontiers in Health Services. 2025;5:1226206.

10.	Johnson K, Posner SF, Biermann J, Cordero JF, Atrash HK, Parker CS, et al. Recommendations to improve preconception health and Health Care—United States: report of the CDC/ATSDR preconception care work group and the select panel on preconception care. Morbidity and Mortality Weekly Report: Recommendations and Reports. 2006;55(6):1-CE-4.

11.	Gesesew H, Berhane K, Siraj ES, Siraj D, Gebregziabher M, Gebre YG, et al. The impact of war on the health system of the Tigray region in Ethiopia: an assessment. BMJ Global Health. 2021;6(11).

12.	Tang AM, Chung M, Dong KR, Bahwere P, Bose K, Chakraborty R, et al. Determining a global mid-upper arm circumference cut-off to assess underweight in adults (men and non-pregnant women). Public Health Nutrition. 2020;23(17):3104-13.

13.	Miele MJ, Souza RT, Calderon IdMP, Feitosa F, Leite DF, Rocha Filho E, et al. Proposal of MUAC as a fast tool to monitor pregnancy nutritional status: results from a cohort study in Brazil. BMJ open. 2021;11(5):e047463.

14.	Hayes RJ, Bennett S. Simple sample size calculation for cluster-randomized trials. International journal of epidemiology. 1999;28(2):319-26.

15.	Abneh AA, Getahun WT, Agmassie GA, Gelaw SS, Kassie TD. The magnitude of preconception care utilization and associated factors among women in Ethiopia: systematic review and meta-analysis, 2024. BMC Pregnancy and Childbirth. 2025;25(1):259.

16.	Wegene MA, Gejo NG, Bedecha DY, Kerbo AA, Hagisso SN, Damtew SA. Utilization of preconception care and associated factors in Hosanna Town, Southern Ethiopia. PloS one. 2022;17(1):e0261895.

17.	FMoH GoE. National guideline for the management of acute malnutrition. FMOH Addis Ababa; 2019.

18.	Meles GG, Asgedom AA, Gebretnsae H, Hidru HD, Berhe AK, Gebrekidan GB, et al. Postwar nutritional status of lactating mothers: evidence from war-torn Tigray, Ethiopia. Journal of Health, Population and Nutrition. 2024;43(1):192.

19.	Misgina KH, Boezen HM, Van der Beek EM, Mulugeta A, Groen H. What factors are associated with pre-pregnancy nutritional status? Baseline analysis of the KITE cohort: a prospective study in northern Ethiopia. BMJ open. 2021;11(6):e043484.

20.	Forde C. Scoring the international physical activity questionnaire (IPAQ). University of Dublin. 2018;3.

21.	Haile Mariam D, Estifanos A, Wondimagegn D, Simmons B, Conteh L. Optimizing the Ethiopian health extension programme: strategies to address workforce challenges. 2023.
