# Supplementary material for: Preconception care uptake and risk factors for adverse pregnancy outcomes among pregnant women in Tigray, northern Ethiopia: A community-based cross-sectional study
Source: PLoS One. 2025 Nov 7;20(11):e0336255. doi: 10.1371/journal.pone.0336255 (PMC12594428; doi:10.1371/journal.pone.0336255)
Supplement: Attachment — Submitted filename: Response to Reviewers two.docx [file pone.0336255.s006.docx]

**Date: October 16 2025**

**Manuscript Number:** PONE-D-25-35031

**Title:** **Preconception Care Uptake and Risk Factors for Adverse Pregnancy Outcomes among pregnant women in Tigray, northern Ethiopia: A Community-Based Cross-Sectional Study**

By: Gebremedhin Gebreegziabher Gebretsadik^1, 2*^, Andargachew Kassa  Biratu^4^, Alemayehu Bayray Kahsay ^2^, Amanuel Gessessew ^3^, Zohra S. Lassi, ^5, 6^_,_ Hailemariam Segni^7^, Afework Mulugeta ^2^

To: PLOS one

Dear Editor,

We, the authors of this manuscript, express our gratitude to the journal editors and reviewers for their thorough review, insightful comments, and valuable suggestions that significantly enhanced the quality of our work. We have rigorously revised the manuscript as per your questions and comments. We have included the point-by-point response in the table below, framed as **Editor(s)’and Reviewers' comments** and authors' responses. The detailed revisions and changes we made in the main document are prepared with track changes attached separately. We expect our revision will enable the manuscript to fit the journal better. Should you have any further inquiries, please feel free to contact us at your convenience.

**Point-by-point Responses to Editor’s and Reviewers Comments**

| **Editors and Reviewers' comments** | Authors’ Response |
| --- | --- |
| **Editor comments** |  |
| 1. If the reviewer comments include a recommendation to cite specific previously published works, please review and evaluate these publications to determine whether they are relevant and should be cited. There is no requirement to cite these works unless the editor has indicated otherwise. | Thank you very much for your suggestions. None of the reviewer comments included recommendations to cite specific previously published works. |
| 2. Please review your reference list to ensure that it is complete and correct. If you have cited papers that have been retracted, please include the rationale for doing so in the manuscript text, or remove these references and replace them with relevant current references. Any changes to the reference list should be mentioned in the rebuttal letter that accompanies your revised manuscript. If you need to cite a retracted article, indicate the article’s retracted status in the References list and also include a citation and full reference for the retraction notice. | Thank you for your valuable suggestion. We have checked all reference which included in this manuscript and we did not cite retracted papers |
| **Reviewer #1:** |  |
| 1. Although authors added some information about how they assessed the validity and reliability of instrument for measuring attitude, their answer was not convincing. For instance, they should have report at least CVI, and CVR and Cronbach's alpha. | Thank you for your insightful and valuable feedback. Since the attitude measurement items were not a primary outcome and were used only for descriptive purposes, not in the final logistic regression model, we relied on their use in a prior study conducted in Ethiopia, published in PLoS ONE (***Wegene MA, Gejo NG, Bedecha DY, Kerbo AA, Hagisso SN, Damtew SA. Utilization of preconception care and associated factors in Hosanna Town, Southern Ethiopia. PloS one. 2022;17(1):e0261895***.), as a reference for the validity, without conducting a separate validity test. We appreciate your valuable suggestion. We have incorporated it as a limitation in the Discussion section of the manuscript (page 29, line 527) |
| **Reviewer #2:** |  |
| 1.This abstract introduction effectively highlights a critical global health issue—poor pregnancy outcomes—with a specific focus on low- and middle-income countries. It correctly emphasizes the importance of preconception care (PCC) as a preventive strategy, but also points out a key gap in both implementation and research, especially in conflict-affected regions. The choice to focus on Tigray, Ethiopia, adds relevance and urgency given the area's recent instability. To strengthen this paragraph, the authors might consider briefly defining what PCC includes, or providing a statistic or reference to underscore the magnitude of poor pregnancy outcomes globally. Overall, the paragraph sets a clear and important context for the study. | Thank you very much for your valuable insights. Accordingly, we have revised the background section of the abstract and incorporated these changes into the corresponding section of the manuscript. |

Thanks
